# Allergen Immunotherapy for the Treatment of Eosinophilic Esophagitis. An EAACI Task Force Systematic Review

**DOI:** 10.1002/clt2.70176

**Published:** 2026-06-18

**Authors:** Martina Votto, Georgios Rentzos, Darío Antolin‐Amerigo, Barbara Rymarczyk, George N. Konstantinou, Adam T. Fox, Antonella Cianferoni, Arzu Bakirtas, Carlo Maria Rossi, Marina Tsoumani, Enrico Heffler, Ingrid Terreehorst, Evangelia C. Apostolidou, Maria Beatrice Biló, Oliver Pfaar, Alfredo J. Lucendo, Georgios K. Nikolopoulos, Constantinos Pitsios

**Affiliations:** ^1^ Maternity and Pediatric Department Azienda Sanitaria Locale Benevento Italy; ^2^ Department of Internal Medicine and Clinical Nutrition Institute of Medicine Sahlgrenska Academy University of Gothenburg Gothenburg Sweden; ^3^ Section of Allergology Department of Respiratory Medicine and Allergology Sahlgrenska University Hospital Gothenburg Sweden; ^4^ Allergy Department Hospital Universitario Ramón y Cajal Instituto Ramón y Cajal de Investigación Sanitaria (IRYCIS) Universidad de Alcalá Madrid Spain; ^5^ Department of Internal Diseases, Allergology and Clinical Immunology Medical University of Silesia Katowice Poland; ^6^ Department of Allergy and Clinical Immunology 424 General Military Training Hospital Thessaloniki Greece; ^7^ Department of Paediatric Allergy Evelina London Children's Hospital London UK; ^8^ The Children's Hospital of Philadelphia University of Pennsylvania Medical School Philadelphia Pennsylvania USA; ^9^ Department of Pediatric Allergy and Immunology Faculty of Medicine Gazi University Ankara Türkiye; ^10^ First Department of Internal Medicine Fondazione IRCCS Policlinico San Matteo Pavia Italy; ^11^ Allergy Centre Wythenshawe Hospital Manchester University Hospitals NHS Foundation Trust Manchester UK; ^12^ School of Biological Sciences Faculty of Biology, Medicine and Health University of Manchester Manchester UK; ^13^ Department of Biomedical Sciences Humanitas University Pieve Emanuele Italy; ^14^ Personalized Medicine, Asthma and Allergy IRCCS Humanitas Research Hospital Rozzano (MI) Italy; ^15^ Department of Otorhinolaryngology and Head & Neck Surgery Amsterdam University Medical Center University of Amsterdam Amsterdam the Netherlands; ^16^ Allergy and Clinical Immunology Private Practice Thessaloniki Greece; ^17^ Department of Clinical and Molecular Sciences Polytechnic University of Marche Ancona Italy; ^18^ Allergy Unit Department of Internal Medicine University Hospital of Marche Ancona Italy; ^19^ Department of Otorhinolaryngology, Head and Neck Surgery Section of Rhinology and Allergy University Hospital Marburg Philipps‐Universität Marburg Germany; ^20^ Department of Gastroenterology Hospital General de Tomelloso Tomelloso Spain; ^21^ Centro de Investigación Biomédica en Red en Enfermedades Hepáticas y Digestivas Madrid Spain; ^22^ Medical School University of Cyprus Nicosia Cyprus

**Keywords:** allergen immunotherapy, eosinophilic esophagitis, epicutaneous immunotherapy, subcutaneous immunotherapy

## Abstract

**Background:**

Multiple routes of allergen immunotherapy (AIT) are approved for several IgE‐mediated allergic diseases; however, the use of AIT in eosinophilic esophagitis (EoE) remains controversial and is supported by limited evidence. This review, conducted within the frame of an EAACI Task Force, aims to systematically evaluate the use of AIT as a potential treatment for EoE.

**Methods:**

The protocol was registered and prepared in accordance with PRISMA guidelines. The literature search was conducted across three online databases (PubMed, Embase, and Scopus) and included studies published through January 31^st^, 2025. Risk of Bias was assessed for each eligible study.

**Results:**

Four articles met the inclusion criteria. Three articles evaluated EPIT for milk‐induced EoE in pediatric patients, all from the SMILEE (Study of Efficacy and Safety of Viaskin Milk for milk‐induced EoE) trial and its extensions. These included a randomized, placebo‐controlled trial, its open‐label extension, and a pilot immunological study. The SMILEE trial found no statistically significant difference in tissue eosinophilia between the active (EPIT) and control (placebo) arms in the intention‐to‐treat population, while 47% of treated EoE patients tolerated milk without recurrence of esophageal eosinophilia. This finding was further supported by a subsequent open‐label study with a 2‐year follow‐up. In the third publication, the researchers found that EPIT was associated with decreased Th2‐related transcripts and increased regulatory T‐cell‐associated transcripts. Only one eligible study evaluated the use of SCIT for treating EoE. It was a retrospective case‐control study reporting that SCIT had a neutral effect and yielded inconclusive findings regarding the course of EoE.

**Conclusion:**

There is insufficient high‐quality evidence to support the effectiveness of alternative routes of AIT for the treatment of EoE, either as an add‐on or a standalone treatment.

## 1 Background

Eosinophilic esophagitis (EoE) is a chronic inflammatory disease characterized by symptoms of esophageal dysfunction and histologically by eosinophil‐predominant inflammation [1]. EoE is a type 2 helper cell (Th2)–mediated disease that shares pathophysiologic features with other Th2 disorders and usually develops in atopic patients [2, 3, 4]. As such, EoE patients have been reported to have an increased risk of comorbid respiratory allergies, including asthma, compared with the general population [5]. However, EoE also exhibits distinct Th2‐driven mechanisms restricted to the esophageal mucosa, involving the localized release of epithelial alarmins and type 2 cytokines, as well as eosinophil recruitment [4].

EoE appears to be a multifactorial disease, driven by a combination of genetic predisposition, epithelial barrier dysfunction, environmental risk factors, and allergen sensitization [4, 6]. Although allergy tests to food allergens have not been shown to be useful diagnostic tools for EoE, food remains the most common trigger of the disease, as evidenced by the effectiveness of elimination diets and by the observation that EoE is a potential side effect of oral immunotherapy (OIT) with foods, with an estimated incidence of 2.3% [7, 8, 9].

On the other hand, the role of airborne allergens in triggering EoE has also been described in a few studies, including animal models, case reports, and case series which describe variation in EoE symptoms during pollen seasons, a seasonal increase in newly diagnosed EoE cases, and a higher incidence of food impactions during specific seasons [10, 11, 12, 13]. However, when strict definitions of EoE flares are applied, seasonal exacerbations are infrequently reported, making the evidence for a seasonal effect on EoE contradictory [14]. Furthermore, only anecdotal case reports have described a causal link between EoE occurrence and sublingual immunotherapy (SLIT) with aeroallergens [7].

Although data on the pathophysiological role of allergens in EoE are contradictory, it could be hypothesized that targeting culprit allergens during the treatment of concomitant allergic diseases (such as allergic rhinitis, asthma, or food allergy) through allergen immunotherapy (AIT)‐induced desensitization could have a beneficial effect on EoE, possibly contributing to the prevention of disease onset or a reduction in disease flares [13]. The use of subcutaneous immunotherapy (SCIT) with aeroallergens for the treatment of EoE has not been extensively described, and the available evidence is largely limited to case series and case reports [15, 16, 17, 18, 19]. Moreover, data on the use of epicutaneous immunotherapy (EPIT) with food allergens in patients with EoE are also limited [20].

To clarify the complex relationship between EoE and AIT, a Task Force (TF) has been established within the European Academy of Allergy and Clinical Immunology (EAACI). The outcomes of a systematic review and meta‐analysis conducted by this TF demonstrated that EoE is a potential side effect of OIT with food allergens, whereas it is uncommon during SLIT with aeroallergens [7]. The present TF report presents the findings of a systematic review evaluating the use of AIT as a potential therapeutic approach for EoE.

## 2 Methods

### 2.1 Study Design

The protocol for this review was registered in the International Prospective Register of Systematic Reviews (PROSPERO ID registration: CRD42023427724) and reported in accordance with the Preferred Reporting Items for Systematic Reviews and Meta‐Analyses (PRISMA) [21]. The methodology was reviewed and approved by the authors during remote and in‐person TF meetings.

### 2.2 Search Strategy

The literature search was conducted using three online databases: PubMed, Scopus, and Embase. An expert in systematic reviews (GKN), in consultation with the other authors, helped develop the PubMed search strategy and adapted it for the other databases. All databases were searched from inception to January 31^st^, 2025. Grey literature (e.g., conference abstracts) was also searched, and the reference list of full‐text articles was screened to identify additional relevant studies. The following Medical Subject Heading (MeSH) terms and text words were used in the queries: “eosinophilic esophagitis” OR “eosinophilic oesophagitis” OR “EoE”, combined with (“AND”) “Allergen immunotherapy” OR “Specific immunotherapy” OR “Subcutaneous Immunotherapy” OR “SCIT” OR“desensitization” OR “AIT” OR “Sublingual immunotherapy” OR “SLIT” OR “oral immunotherapy” OR “Epicutaneous immunotherapy”.

### 2.3 Eligibility Criteria

Studies were included in the review according to the Population, Intervention, Comparator, and Outcome (PICO) criteria:

- Population: Human participants with EoE, with no restrictions on age, gender, or geographic origin.
- Intervention(s)/Exposure(s): Any type of AIT administered to patients with diagnosed EoE. Eligible studies included any form of AIT reporting histological or clinical outcomes in patients with EoE. Interventions were considered whether AIT was administered specifically for EoE or to treat concurrent respiratory or food allergies, provided that EoE‐specific outcomes were documented.
- Comparator(s)/Control(s): No restriction was applied to comparator type. Controlled studies, as well as longitudinal studies without a control group comparing EoE assessment before and after AIT, were considered eligible. For studies on SCIT, comparators could also include parallel patient groups receiving AIT for seasonal aeroallergens (e.g., pollen) versus perennial aeroallergens (e.g., house dust mites or animal dander).
- Main Outcome(s)/Additional outcome(s): Two related yet distinct research questions were addressed, both focusing on the use of AIT in the treatment of EoE. The first evaluated the efficacy of AIT in patients with EoE sensitized to aeroallergens, receiving AIT directed against the relevant inhalant allergens. The second assessed the treatment of EoE using food desensitization approaches, including EPIT or other AIT routes.

Inclusion criteria: Observational (prospective and retrospective) and interventional studies with no restriction on publication languages, published in peer‐reviewed journals, and conducted in humans examining the use of AIT as a treatment option for EoE, were considered eligible.

Exclusion criteria: Case reports, case series, narrative reviews, opinion articles, and editorials were excluded. Studies conducted in animals, as well as in vitro or ex vivo studies that did not directly report clinical EoE outcomes (e.g., EoE symptoms or histological findings), were also excluded. Studies evaluating interventions other than AIT, such as food supplements or herbal infusions, were not considered (Table 1).

**TABLE 1:** Inclusion and exclusion criteria.

Inclusion criteria	Exclusion criteria
Observational (prospective and retrospective) and interventional studies examining AIT for the treatment of EoE in humans.	Case reports, case series, reviews, meta‐analyses, cohorts, opinion articles, editorials. Studies on animals. In vitro or ex vivo studies not directly referring to clinical data (EoE symptoms). Studies referring to other procedures, for example, the use of food supplements or herbal infusions. Studies on EoE caused by AIT.

Abbreviations: AIT, allergen immunotherapy; EoE, eosinophilic esophagitis.

### 2.4 Study Selection, Data Extraction, and Risk of Bias Assessment

Search results were uploaded to Mendeley and deduplicated. Literature citations were imported into the Rayyan web tool, and two investigators (G.R. and B.R.) independently assessed the eligibility of all identified abstracts [22, 23]. Discrepancies were resolved through discussion, and a third reviewer (M.V.) was consulted. Full copies were obtained.

Two authors (G.R and M.V.) independently extracted data from the primary studies using a standardized, detailed data sheet that included the first author's last name, publication year, study type, allergen, type of AIT, population characteristics (sample size and age group), and outcomes, with particular emphasis on the number of cases achieving EoE remission.

Risk of Bias (RoB) was assessed for each eligible study. Study quality was evaluated based on the reporting of patient demographic data, AIT modality, clinical and histopathological diagnostic criteria for EoE, treatment outcomes, and study design, as described in our previous TF publications [7, 24]. In non‐randomized intervention studies, ROBINS‐I was used to assess RoB across seven domains [25]. For randomized controlled trials (RCTs), the Cochrane Collaboration RoB2 Tool was used to assess RoB across five domains [26].

### 2.5 Data Analysis and Synthesis

Detailed information on the included studies is presented in tables for each question, describing study design and participants (number and age groups), AIT route, and outcomes. Due to the limited number of included articles, a meta‐analysis was not performed. Instead, a narrative synthesis of the data was undertaken.

## 3 Results

### 3.1 Summary of Included Studies

**FIGURE 1:**
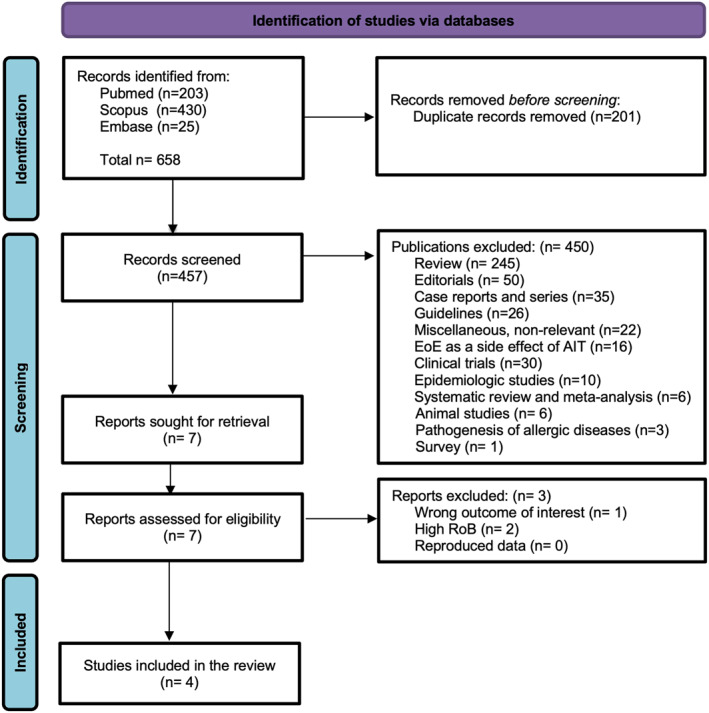
PRISMA flow chart.

The systematic search identified 658 articles, of which 201 were duplicates. Of the 457 retrieved articles, 7 were eligible for full‐text assessment. After screening, 4 articles met the inclusion criteria. The PRISMA flow diagram outlines the screening and selection process (Figure 1). All included studies were conducted in the USA between 2019 and 2020.

Three articles evaluated EPIT for milk‐induced EoE in children and adolescents, all from the SMILEE (Study of Efficacy and Safety of Viaskin Milk for milk‐induced EoE) trial and its extensions. These included a randomized, placebo‐controlled trial, its open‐label extension, and a pilot immunological study. Only one eligible article evaluated the use of SCIT for EoE treatment, and it was a retrospective case‐control study (Table 2).

**TABLE 2:** Summary of included articles.

Author, year	Study design	AIT route	Allergen(s)	Population	Outcomes
Spergel et al. (2020) [20]	Randomized, placebo‐controlled trial with an open‐label extension	EPIT	Milk	20 children (4–17 years old) with milk‐induced EoE Viaskin milk (*n* = 15) Placebo (*n* = 5)	In the intent to treat population, there was no significant difference between the arms in mean eos/hpf. In the per‐protocol population, patients given Viaskin milk had a significantly lower mean eos/hpf count. At study completion, 47% of patients who continued open‐label Viaskin milk had histologic remission.
Spergel et al. (2021) [27]	Open label trial (follow‐up)	EPIT	Milk	19 children (4–17 years old) with milk induced EoE treated with Viaskin milk	Four of five subjects who had < 6 eos/hpf after milk introduction were able to continue with approximately 2 servings of CM/day without any symptoms. One child had an upper EGD with 0 eos/hpf. Two subjects, who had 6–14 eos/hpf during the study, continued to tolerate CM. In addition, 4 subjects who had significant symptoms ingesting CM and had > 15 eos/hpf during the initial SMILEE study were able to add CM back into their diet without having symptoms with concomitant swallowed steroids therapy.
Ruffner et al., 2021 [28]	Study on immunological effect of EPIT in EoE patients.	EPIT	Milk	15 children with milk induced EoE treated with Viaskin milk in the SMILEE trial.	EPIT therapy is associated with significant enrichment in pathways related to T‐cell receptor signaling, antigen presentation and costimulation, and cytokine signaling, as well as upregulation of genes associated with regulatory T‐cell function.
Robey et al. (2019) [29]	Retrospective cohort study (database revision according to ICD9 codes)	SCIT	House dust mites, cat, dog, cockroach, trees mix, grasses mix, weeds mix, Ragweed, English Plantain, and Alternaria	10 EoE + SCIT patients, 9 of whom received the EoE diagnosis before SCIT versus 667 EoE‐SCIT patients	Only 5 patients had long‐term follow‐up data: 40% presenting a histological response and 40% a symptom response. No firm conclusions on the impact on treatment outcomes, due to the limited number of EoE + SCIT.

Abbreviations: AIT, allergen immunotherapy; CM: cow's milk, EGD: esophagogastroduodenoscopy; EoE, eosinophilic esophagitis; eos/hpf, eosinophils per high power field; EPIT, epicutaneous immunotherapy; SCIT, subcutaneous immunotherapy.

### 3.2 EPIT for Treating EoE

Data on the use of EPIT for the treatment of EoE were derived from the SMILEE study. This study enrolled 20 children (ages 4–17 years) with endoscopically confirmed milk‐induced EoE. Fifteen patients were randomized to receive active treatment with Viaskin milk, while 5 received placebo for 9 months [20]. After the treatment period, the authors found no significant difference between the groups in histologic remission upon reintroduction of cow's milk (the primary endpoint) in the intention‐to‐treat population. However, within the per‐protocol population, there was a −69.37 (95% CI, −117.47 to −21.28) least squares means difference between the active‐treatment (*n* = 7 patients) and the placebo (*n* = 2 patients) group. At study completion, 47% of patients who continued open‐label Viaskin milk for an additional 11 months had mean eosinophil counts of fewer than 15 eosinophils per high‐power field (eos/hpf). The same research group published results from a 2‐year follow‐up of the open‐label phase, involving 19 of the previously enrolled children [27]. They found that most patients in the responder (< 6 eos/hpf) and partial responder (6–14 eos/hpf) groups remained symptom‐free while consuming an average of 480 mL/day of cow's milk, indicating that the effect of EPIT for milk can persist for two years after stopping immunotherapy.

In a third publication from the same group, they evaluated the immunological effects of EPIT in 15 of 19 children enrolled in the SMILEE trial, analyzing transcriptional changes in the peripheral CD4^+^ T‐cell compartment during active EoE and after EPIT administration [28]. According to the study results, EPIT to milk altered the peripheral CD4^+^ T‐cell responses in patients with EoE, with significant enrichment in pathways related to T‐cell receptor signaling, antigen presentation and co‐stimulation, and cytokine signaling, as well as upregulation of genes associated with regulatory T‐cell function.

### 3.3 SCIT for Treating EoE

In the systematic review, only one eligible study examined the effect of SCIT in treating EoE. This retrospective cohort study used a clinicopathologic database and included patients across all age groups [29]. ICD‐9/10 codes identified patients with EoE and those treated with SCIT for various aeroallergens. Confirmed EoE patients were cross‐referenced with SCIT‐treated patients to define the EoE + SCIT (EoE patients receiving SCIT) and EoE‐SCIT (EoE patients not receiving SCIT) groups.

A total of 10 EoE + SCIT and 667 EoE‐SCIT patients were identified. At the first follow‐up endoscopy, 3/10 EoE + SCIT patients showed a histologic response, and 6/10 reported symptom improvement. Of the 5 patients with long‐term follow‐up data, 2 achieved histologic remission and 2 experienced symptom improvement. Importantly, in the EoE + SCIT group, there was no reduction in histologic response rates or worsening of overall symptom responses. Overall, the authors concluded that SCIT had a neutral effect on the course of EoE, neither improving nor impairing histologic outcomes.

## 4 Discussion

This study is the first systematic review examining the therapeutic effects of AIT in the management of EoE. According to this study's results, there is insufficient support of the effectiveness of AIT in the treatment of EoE, whether as an add‐on therapy or a standalone treatment.

EPIT is a non‐oral, non‐injectable investigational treatment currently in late‐phase clinical development for peanut and milk allergies, demonstrating good efficacy and a strong safety profile across multiple Phase 2 and 3 studies [30]. Evidence on EPIT for EoE remains extremely limited, consisting of a single small pediatric trial and its open‐label extension [20, 27]. Although the per‐protocol analysis showed a statistically significant improvement in histologic response compared with placebo, the very small sample size substantially limits the generalizability of these findings. Furthermore, results from the 2‐year follow‐up study of EPIT with milk were largely based on esophageal symptom assessment, with histologic evaluation performed in only a limited number of patients, representing a major methodological limitation. Taken together, these factors currently preclude firm conclusions regarding the efficacy of EPIT in the treatment of EoE. The immunological mechanism of action of EPIT appears to involve a decrease in the average expression of Th2‐associated transcripts and an increase in the average expression of regulatory T‐cell‐associated transcripts [28].

The SMILEE trials had several limitations. First, they were pilot studies with small sample sizes, limiting the generalizability of the findings. The authors reported that patients' adherence to a cow's milk‐free diet was not always strict, which may partly explain the unsatisfactory results in the intention‐to‐treat population analysis. These data may also suggest that EPIT provides only partial, rather than complete, tolerance to milk, possibly due to the age of the patients studied (patches have been shown to be more effective in children younger than 12 years) or the short duration of the study (1 year vs. 3 years used in other clinical trials).

The use of SCIT showed neutral and inconclusive outcomes in the retrospective case‐control study included in our systematic review. The findings are limited by the fact that some patients received corticosteroids and dietary interventions in addition to SCIT. Moreover, the proportion of endoscopically dilated patients in the series of 10 treated patients was considerable (30%) and higher than in the control group (21%), which may have positively influenced the study results, but a structured symptom score was not used.

Beyond the study by Robey et al., we identified additional literature exploring the safety and efficacy of SCIT for the treatment of EoE. A controlled study, published as a congress abstract, reported SCIT use in 39 EoE patients sensitized to airborne and food allergens, combined with a component‐resolved diagnostics (CRD)‐based elimination diet [17]. Clinical improvement was reported, with 68% of patients discharged from follow‐up, defined as negative biopsy findings, absence of symptoms, and no need for medications. However, this report lacked sufficient methodological detail and essential information regarding patient selection. Due to substantial missing data, it was excluded from the systematic review.

In a longitudinal study, Armentia et al. investigated whether EoE shares a similar pathological mechanism with pollen‐induced asthma, using CRD to identify cross‐reactive proteins and guide targeted treatments, including food elimination diet and AIT. The study enrolled 129 patients with EoE and 3 control groups (50 patients with allergic asthma, 50 healthy controls, and 53 patients with celiac disease). They found that after 2 years of a CRD‐guided elimination diet and/or AIT (SCIT and SLIT), 78% of EoE patients showed significant clinical improvement, and 75% were in remission (absence of symptoms and negative endoscopy) [31]. Another publication from the same authors reported the use of SCIT in patients with EoE, with or without a CRD‐based elimination diet [32]. The authors reported a 74% remission rate of EoE, defined by negative biopsies, absence of symptoms, no medication use, and no symptom relapse. The same group has recently published a further report on this cohort without providing any additional methodological or clinical information [33]. Across these publications, key data required for systematic evaluation were insufficiently reported, including an insufficient description of how patients received treatments and incomplete reporting of patient follow‐up. Given the incomplete presentation of results and the limited information available for independent evaluation, these studies were excluded from the systematic review.

The role of SCIT in the treatment of EoE has also been described in case reports (Table 3). Ramirez et al. described a 4‐year‐old boy with refractory EoE who responded both histologically and symptomatically to SCIT for dust mites [16]. De Swert et al. reported a 10‐year‐old boy who experienced symptomatic and histologic improvement after SCIT desensitization to grass and birch allergens [15]. Iglesia et al. reported a 14‐year‐old boy with EoE who achieved sustained remission following multiallergen SCIT treatment [34]. Castilano et al. described a 30‐year‐old male who experienced symptom resolution and normalization of esophageal biopsies after SCIT against dust mites, cockroaches, weeds, and trees [18]. Notably, a 10‐year‐old boy experienced exacerbation of EoE symptoms following SLIT and SCIT to grass pollen, suggesting a potential risk associated with AIT in EoE [19].

**TABLE 3:** Case reports on the use of SCIT for EoE.

Author, year	AIT route	Allergen(s)	Population	Outcomes
De Swert et al. (2013) [15]	SCIT	Bet v 1 and Phl p 1	10‐year‐old boy with allergic rhinitis and EGID	After 3 years of SCIT, he had only minor complaints of rhinoconjunctivitis and gastrointestinal symptoms and he was able to reintroduce fresh apple into his diet without a relapse of symptoms.
Ramirez and Jacobs et al. (2013) [16]	SCIT	Dust mite	4‐year‐old boy with allergic rhinitis and EoE	Before AIT, the patient was tried on FED then oral fluticasone + PPI without histologic remission. After AIT he was able to stop fluticasone and continued to receive PPI for about 6 months before he had an esophageal biopsy, which revealed histologic remission. At the age of 8 years, the patient continued to be in remission.
Castilano et al. (2013) [18]	SCIT	Dust mites, cockroaches	30‐year‐old male	Despite treatment with oral fluticasone for 2 years, the patient had persistent dysphagia requiring multiple esophageal dilations. Skin testing identified reactions to dust mites, cockroaches, common weeds and trees. The patient received immunotherapy to these aeroallergens without any relapse in dysphagia or food impaction. Esophageal biopsy showed complete resolution of eosinophil infiltration.
Wells et al. (2018) [19]	SLIT than SCIT	Grass pollen	A 10‐year‐old boy with nut allergy, allergic rhinitis, asthma and quiescent EoE.	The child developed recurrence of EoE symptoms on two separate occasions, coincident with the commencement of SLIT then SCIT to grass pollen.
Iglesia et al. (2021) [33]	Multiallergen SCIT	Vial 1: Mites, trees, Grasses Vial 2: Cat, Dog, weeds, Mold	14‐year‐old male	The remission was maintained over 18 months of maintenance SCIT.

Abbreviations: AIT, allergen immunotherapy; EGID, eosinophilic gastrointestinal disorders; EoE, eosinophilic esophagitis; FED, food elimination diet; PPI, proton pump inhibitor; SCIT, subcutaneous immunotherapy; SLIT, sublingual immunotherapy.

The limited number of retrieved articles precluded a meta‐analysis. Moreover, the included articles had several limitations, including small sample sizes and heterogeneous study designs.

**FIGURE 2:**
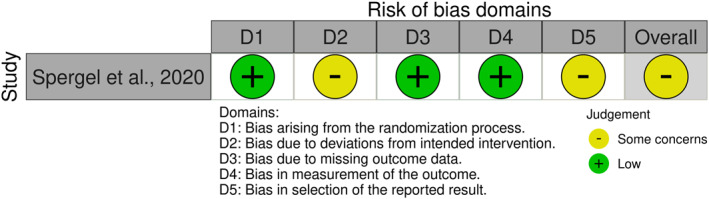
Risk of bias (RoB) domain for retrieved randomized trial (RoB2 tool).

**FIGURE 3:**
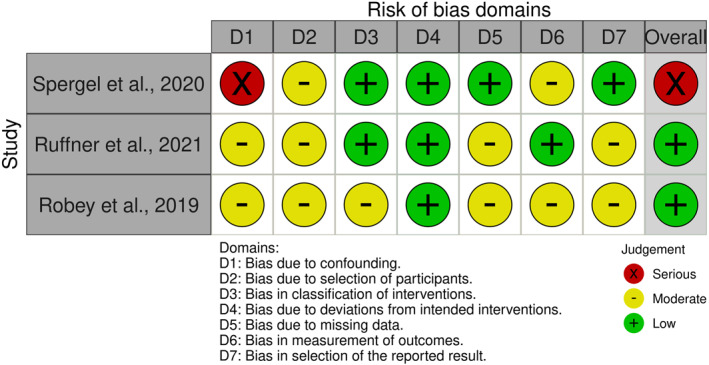
Risk of bias (RoB) domain for retrieved observational studies (ROBINS‐I v2 tool).

The studies included in this systematic review show an overall moderate risk of bias (Figures 2 and 3). This is mainly due to small sample sizes, differences in study design, and methodological issues such as lack of blinding and incomplete randomization. Additional factors, including retrospective data collection, uncontrolled confounders, and selective reporting, further limit the internal validity of the results. Therefore, the current evidence on the effectiveness of AIT for EoE should be interpreted with caution. Well‐designed, adequately powered randomized controlled trials are needed to provide more reliable conclusions.

## 5 Conclusion

This systematic review found limited high‐quality evidence supporting the use of AIT for treating EoE, beyond its established role in IgE‐mediated allergies. EPIT showed signals of potential benefit in the SMILEE study, while observational reports and case studies of SCIT suggest potential improvement, underscoring the heterogeneous response to these interventions. At present, AIT cannot be recommended as either standard or add‐on therapy for EoE. Overall, current evidence supports a cautious, individualized approach to AIT in EoE, integrating clinical judgment, patient preferences, and careful monitoring of disease activity.

## Author Contributions

**Martina Votto:** writing – original draft, writing – review and editing, conceptualization. **Georgios Rentzos:** writing – original draft, writing – review and editing, methodology, conceptualization. **Darío Antolin‐Amerigo:** writing – review and editing, conceptualization. **Barbara Rymarczyk:** writing – review and editing, supervision, methodology. **George N. Konstantinou:** writing – review and editing, supervision. **Adam T. Fox:** writing – review and editing, supervision. **Antonella Cianferoni:** writing – review and editing, supervision. **Arzu Bakirtas:** writing – review and editing. **Carlo Maria Rossi:** writing – review and editing. **Marina Tsoumani:** writing – review and editing. **Enrico Heffler:** writing – review and editing. **Ingrid Terreehorst:** writing – review and editing, supervision. **Evangelia C. Apostolidou:** writing – review and editing. **Maria Beatrice Biló:** writing – review and editing, supervision. **Oliver Pfaar:** writing – review and editing, supervision. **Alfredo J. Lucendo:** supervision, writing – review and editing. **Georgios K. Nikolopoulos:** methodology, data curation, writing – review and editing. **Constantinos Pitsios:** conceptualization, supervision, writing – review and editing, writing – original draft.

## Ethics Statement

This study was supported by the European Academy of Allergy and Clinical Immunology (EAACI) as part of the Task Force “Eosinophilic esophagitis and allergen immunotherapy” (Budget code 40806); years 2022–2024.

## Conflicts of Interest

The authors declare no conflicts of interest.

## Data Availability

Data sharing not applicable to this article as no datasets were generated or analysed during the current study.
